# Supplementary material for: A rapid qualitative methods assessment and reporting tool for epidemic response as the outcome of a rapid review and expert consultation
Source: PLOS Glob Public Health. 2023 Oct 27;3(10):e0002320. doi: 10.1371/journal.pgph.0002320 (PMC10610454; doi:10.1371/journal.pgph.0002320)
Supplement: S1 File — (DOCX) [file pgph.0002320.s001.docx]

**Supporting Information File 1:** Search Strings in all languages

| **Language** | **Search Terms/String** |
| --- | --- |
| **English** | (((ethnography OR virtual ethnography OR anthropology OR fieldwork OR participant observation OR video-based observation OR qualitative observation OR ethnographic observation OR  interview OR mixed methods OR multi-method OR fieldnotes OR focus group OR participatory OR community based OR multi-sector initial rapid assessment OR qualitative OR digital qualitative research OR delphi OR group discussion OR case study OR documentary) AND (research OR study OR review OR assessment OR evaluation OR appraisal OR inquiry OR analysis OR review OR feedback OR data collection OR assessment procedure OR assessment response OR assessment evaluation)) AND (rapid OR quick OR short-term OR short-duration)) AND ((emergency OR emergency relief OR emergency response OR epidemic OR pandemic OR disease outbreak OR outbreak OR response OR crisis OR infectious disease OR disaster OR humanitarian) AND health)) |
| **French** | (« ethnographie » OU « ethnographie virtuelle » OU « anthropologie » OU « travail de terrain » OU « observation participante » OU « observation vidéo » OU « observation qualitative » OU « observation ethnographique » OU « entretien ou méthodes mixtes » OU « multi-méthodes » OU « notes de terrain » OU « Focus groupe » OU « participatif » OU « communautaire » OU « évaluation rapide initiale multisectorielle » OU « qualitatif » OU « recherche qualitative digitale » OU « delphi » OU « groupe de discussion » OU « étude de cas » OU « documentaire » ET (« recherche » OU « étude » OU « revue » OU « appréciation » OU « évaluation » OU « estimation » OU « enquête » OU « analyse » OU « retour d’information » OU « collecte de données » OU « procédure d’évaluation » OU « réponse d’évaluation » OU « évaluation » ET (« rapide » OU « accéléré » OU « court » OU « court terme » ET (« urgence » OU « secours d’urgence » OU « réponse aux urgences » OU « épidémie » OU « pandémie » OU « urgence » OU « foyer épidémique » OU « émergence sanitaire » OU « épidémie » OU « réponse » OU « riposte » OU « crise » OU « maladie infectieuse » OU « désastre » OU « humanitaire » ET (« santé »))))) |
| **Mandarin** | ((((民族志 OR 虚拟民族志 OR 人类学 OR 田野 OR 参与式观察 OR 视频观察OR 定性观察OR 民族志观察 OR 访谈 OR 混合方法 OR 多元方法 OR 田野笔记 OR 焦点小组 OR 参与式 OR 基于社区的OR 多部门初步快速评估 OR 定性OR 数字化定性研究 OR 德尔菲法 OR 小组讨论 OR 个案研究 OR 纪录片) AND (研究 OR 回顾OR 评定 OR 评价 OR 评估 OR 探究 OR 分析 OR 反馈 OR 数据搜集OR 评估程序OR 评估回应OR 评估评价)) AND (快速的 OR 快的 OR 短期的 OR 持续时间短的)) AND ((紧急事件 OR 紧急救援OR 应急反应OR 疫情 OR 大流行 OR 疾病爆发OR 爆发 OR 反应 OR 危机 OR 传染病 OR 灾难 OR 人道主义) AND 健康)) |
| **Portuguese** | ((((ethnography OR virtual ethnography OR anthropology OR fieldwork OR participant observation OR video-based observation OR qualitative observation OR ethnographic observation OR  interview OR mixed methods OR multi-method OR fieldnotes OR focus group OR participatory OR community based OR multi-sector initial rapid assessment OR qualitative OR digital qualitative research OR delphi OR group discussion OR case study OR documentary) AND (research OR study OR review OR assessment OR evaluation OR appraisal OR inquiry OR analysis OR review OR feedback OR data collection OR assessment procedure OR assessment response OR assessment evaluation)) AND (rapid OR quick OR short-term OR short-duration)) AND ((emergency OR emergency relief OR emergency response OR epidemic OR pandemic OR disease outbreak OR outbreak OR response OR crisis OR infectious disease OR disaster OR humanitarian) AND health)) |
| **Spanish** | ((((ethnography OR virtual ethnography OR anthropology OR fieldwork OR participant observation OR video-based observation OR qualitative observation OR ethnographic observation OR  interview OR mixed methods OR multi-method OR fieldnotes OR focus group OR participatory OR community based OR multi-sector initial rapid assessment OR qualitative OR digital qualitative research OR delphi OR group discussion OR case study OR documentary) AND (research OR study OR review OR assessment OR evaluation OR appraisal OR inquiry OR analysis OR review OR feedback OR data collection OR assessment procedure OR assessment response OR assessment evaluation)) AND (rapid OR quick OR short-term OR short-duration)) AND ((emergency OR emergency relief OR emergency response OR epidemic OR pandemic OR disease outbreak OR outbreak OR response OR crisis OR infectious disease OR disaster OR humanitarian) AND health)) |
