# Supplementary material for: A rapid qualitative methods assessment and reporting tool for epidemic response as the outcome of a rapid review and expert consultation
Source: PLOS Glob Public Health. 2023 Oct 27;3(10):e0002320. doi: 10.1371/journal.pgph.0002320 (PMC10610454; doi:10.1371/journal.pgph.0002320)
Supplement: S2 File — (DOCX) [file pgph.0002320.s002.docx]

**Supporting Information File 2: First round data extraction template for rapid qualitative methods**

**Full bibliographic reference:**

**Source (name of database; gray literature; manual inclusion):**

**Summary of tool** ($\cong$*10 lines*)**:**

| **Evaluation criteria** | **Reviewer notes** |
| --- | --- |
| **Qualitative tool used** |  |
| **Data collected** |  |
| **Time frame for conduct & analysis** |  |
| **Training (type, ease of)** |  |
| **Recruitment procedure** (How research subjects/participants are recruited) |  |
| **Target group**  Refers to   1. Subjects/participants of the study 2. The target population that the study plans to “intervene” or “help” or “influence” |  |
| **Applicability to more than one group** |  |
| **Applicability for vulnerability** (context and case specific, not limited by pre-existing definitions) |  |
| **Single or multiple site** |  |
| **Community participation** |  |
| **Restitution (to community or other actors)**  (whether the results of the study will be restituted to the community being studied or other social groups) |  |
| **Ease of data collection** |  |
| **Ease of analysis** |  |
| **Reporting guideline** |  |
| **Other advantages noted** |  |
| **Other disadvantages noted** |  |
| **Material and human resources requirement**  **(if available)** |  |
| **Estimated cost**  **(if available)** |  |
| **(Potential) Impact of study** | *Citations* |
|  | *Reported impact* |
|  | *Potential impact* |
| **Applicability to low-resource settings** |  |
| **Ethical concerns** |  |
|  |  |

**Overall quality of method and its tools** (textual assessment of the quality of the methodology, tools used, results)
