# Supplementary material for: A rapid qualitative methods assessment and reporting tool for epidemic response as the outcome of a rapid review and expert consultation
Source: PLOS Glob Public Health. 2023 Oct 27;3(10):e0002320. doi: 10.1371/journal.pgph.0002320 (PMC10610454; doi:10.1371/journal.pgph.0002320)
Supplement: S3 File — (DOCX) [file pgph.0002320.s003.docx]

**Supporting Information File 3: Second round coding of rapid qualitative methods**

**Full bibliographic reference:**

**Source (name of database; gray literature; manual inclusion):**

**Summary of tool** ($\cong$*10 lines*)**:**

| **Evaluation criteria** | **Reviewer notes** | **Points** |
| --- | --- | --- |
| **Qualitative tool used** |  | -- |
| **Data collected** |  | -- |
| **Time frame for conduct & analysis** |  | /5 |
| **Training (type, ease of)** |  | /5 |
| **Recruitment procedure** (How research subjects/participants are recruited) |  | -- |
| **Target group**  Refers to   - Subjects/participants of the study - The target population that the study plans to “intervene” or “help” or “influence” |  | -- |
| **Applicability to more than one group**  Yes = 5  No = 0 |  | /5 |
| **Applicability for vulnerability** (context and case specific, not limited by pre-existing definitions)  Yes = 5  No = 0 |  | /5 |
| **Single or multiple site** |  | -- |
| **Community participation** (development, testing, implementation, analysis)  0=no participation at all  1=poor participation  2=average participation  3=good participation  4=very good participation  5=excellent participation |  | /5 |
| **Restitution (to community or other actors)**  (whether the results of the study will be restituted to the community being studied or other social groups)  0 = No restitution  5= Yes, some form of restitution of results |  | /5 |
| **Ease of data collection** |  | /5 |
| **Ease of analysis** |  | -- |
| **Reporting guideline (COREQ, SRQR, etc)**  0 = No reporting  5 = Reporting |  | /5 |
| **Other advantages noted** |  | -- |
| **Other disadvantages noted** |  | -- |
| **Material and human resources requirement**  **(if available)** |  | -- |
| **Estimated cost**  **(if available)** |  | -- |
| **Study impact** | *Citations* (#times cited by other sources)  0 = No citations  3 = Under 5 citations  5 = Over 5 citations | /5 |
|  | *Potential impact*   - - Whether paper could be transformative for current delivery of services   - Whether paper offers insight that could transform institutional understanding of the issue, and thus indirectly transform delivery of services   - Whether paper addressed a known, recognized, and important issue   - Whether paper had enough methodological rigor to be persuasive to non-social science audiences   - Whether paper demonstrated a social science innovation   0 = None of the above  1 = One of the above criteria  2 = Two of the above criteria  3 = Three of the above criteria  4 = Four of the above criteria  5 = Five of the above criteria | /5 |
| **Applicability to low-resource settings**  0 = Not applicable to any low-resource settings  3 = applicable to some low-resource settings (e.g., applicable to middle-to-high income populations in urban cities)  5 = applicable to all low-resource settings. |  | /5 |
| **Ethical concerns** |  |  |
|  | **Total** | /55 |

**Overall quality of method and its tools** (textual assessment of the quality of the methodology, tools used, results)

**Glossary and scoring procedures**

Below is a glossary of the criteria that we will be evaluating numerically. Please note that **Evaluators** are those who score the rapid qualitative methodology. **Reviewers** are those who collected and provided a textual summary of the methodology on the Evaluation Summary form.

**Scoring** is from 0 to 5, with 5 being the best or highest impact and 0 being the least effective. We have kept each criterion to be evaluated at 5 points for ease and consistency.

If one criterion is not scored, the evaluator should simply take into account the notes from the Review teams.

For criteria that are numerically assessed, evaluators will score in two ways:

1. Likert scale:

0=very poor (no impact, very complex, inappropriate)

1=poor (low impact, complex, marginally appropriate)

2=average

3=good

4=very good

5=excellent, effective

1. Yes/no

Some criteria with yes/no answers will be scored with 0 or 5 points only. These are clearly flagged below.

1. **Time frame for conduct & analysis**

This measure speaks to the *rapidity* of the implementation of the tool, from training through data collection and analysis. (Recall that we set as inclusion criteria methods that authors identified as “rapid”, or under six months.)

With this factor, the evaluator is asked to determine whether the time frame for the investigation is appropriate for the emergency at hand. A slow-moving, lengthy epidemic or pandemic may necessitate longer investigation periods, whereas a humanitarian crisis may need rapid study (within a couple of weeks).

The evaluator needs to judge whether the time frame of the method was appropriate, too short, or too long for the emergency at hand.

If the conduct is specified, but the analysis is not, then use the “first submission” date as a reference. (if possible and necessary, may contact the authors for the exact dates as well)

1. **Training**

Here we are evaluating how easy or difficult it is to train field researchers, as well as what the training involves. We may not always have access to how field researchers were trained in which case we can

Training involves two measures:

Timing

If training to conduct the methodology and analyze resulting data requires training over *several weeks*, this feature would be accorded fewer points.

If training can be conducted over *several days to a week* (with ongoing supervision, as needed), it could obtain five points.

Specialization

Does the method require highly specialized training, for instance, in the use of ethnography or in Geographic information system mapping? If so, then the method probably needs to be downgraded, even if is an excellent one, simply because it isn’t something that can be widely used.

Does the training require relatively little specialized training?

1. **Applicability to more than one group**

The evaluation requires answering how the methodology could be used to investigate other social groups or communities, or whether it could be used in other contexts in addition to the one in which the study used it. If not explicitly stated by the author(s), the evaluator should make a judgement on it.

This feature requires a yes/no answer.

Yes = 5

No = 0

1. **Applicability to vulnerability**

Here vulnerability must be understood as context- and case-specific and is not limited by pre-existing definitions of vulnerability.

So, for instance, if researchers used an ethnographic method that is really best used with a single occupational group, then the evaluation would be *No*.

I’m not sure whether this is a yes/no answer or a Likert scale, but am inclined to think that this would be a yes/no: either it’s applicable to studies investigating vulnerability, or it isn’t.

Yes = 5

No = 0

1. **(a) Community participation**

When we refer to community participation, we mean the participation of the study population in any aspect of the development, testing, carrying out, or analysis of the methodology. There may be some overlap with the following measure, on restitution.

This feature would be evaluated with a Likert scale.

0=no participation at all

1=poor participation

2=average participation

3=good participation

4=very good participation

5=excellent participation

5 (b) Stakeholder participation:

Stakeholders refer to formal and/or informal actors at multiple levels and across different sectors. They may overlap with “community”, but they can also be the leaders during the emergencies.

The evaluator may need to take a guess, since sometimes there’s sufficient information in the article.

1. **Restitution to community or other actors**

Restitution to community or other actors is about whether the results of the study are reported to the community being studied, to other social groups, or to authorities involved in the question being studied.

This is a Yes/No question.

0 = No restitution

5= Yes, there is some form of restitution of results

1. **Ease of data collection**
2. **Reporting guideline**

Responses to this question are evaluated according to whether the article, manuscript, or any other version providing results to the study adhere to any reporting guidelines, notably COREQ or SRQR (or equivalent guidelines in contexts other than English), although depending on the study, others could be relevant.

This is a Yes/No question.

0 = No reporting

5 = Reporting

1. **Study impact**

Impact of the study is a central measure by which we evaluate the methodology used. “Impact” is defined as the study’s influence in terms of:

- *Citations*: the social sciences of urgent epidemic situations (or a specific sub-field or discipline within the social sciences).

0 = No citations

3 = Under 5 citations

5 = Over 5 citations

The reason for this scoring is that although we think it is important if a study or its methodology is widely cited, we do not want to bias our evaluation by giving too much weight to older studies. We also think that this measure will be counterbalanced by other measures of impact.

- *Potential influence: in the Evaluator’s opinion, whether the study and methodology stand to have an influence on policy, on adherence to policy or an intervention, or on the formulation or adaptation of an intervention*
  - Whether paper could be transformative for current delivery of services
  - Whether paper offers insight that could transform institutional understanding of the issue, and thus indirectly transform delivery of services
  - Whether paper addressed a known, recognized, and important issue
  - Whether paper had enough methodological rigor to be persuasive to non-social science audiences
  - Whether paper demonstrated a social science innovation

0 = None of the above

1 = One of the above criteria

2 = Two of the above criteria

3 = Three of the above criteria

4 = Four of the above criteria

5 = Five of the above criteria

1. **Applicability to low-resource settings**

Here we are evaluating how easily the methodology can be used in a low-resource setting. If a specific tool requires, for instance, constant internet connection, smartphone access, or other resources, it will not be appropriate for low-resource settings.

I think we could score this as a Yes/No response, simply because it would be too difficult to get into the details of what is needed.

0 = Not applicable to any low-resource settings

3 = applicable to some low-resource settings (e.g., applicable to middle-to-high income populations in urban cities)

5 = applicable to all low-resource settings.
