# Supplementary material for: A rapid qualitative methods assessment and reporting tool for epidemic response as the outcome of a rapid review and expert consultation
Source: PLOS Glob Public Health. 2023 Oct 27;3(10):e0002320. doi: 10.1371/journal.pgph.0002320 (PMC10610454; doi:10.1371/journal.pgph.0002320)

**Supporting Information File 4: Language-specific data extraction flowcharts and notes**

**
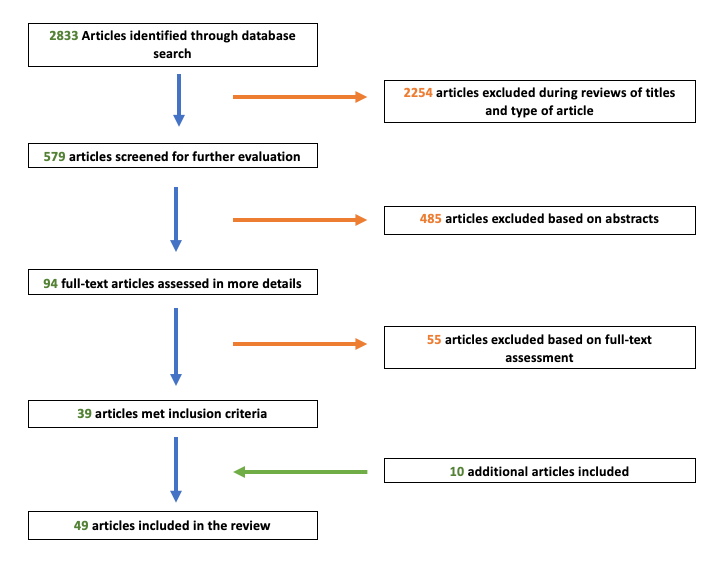
**

Mandarin Language Data Extraction Flowchart


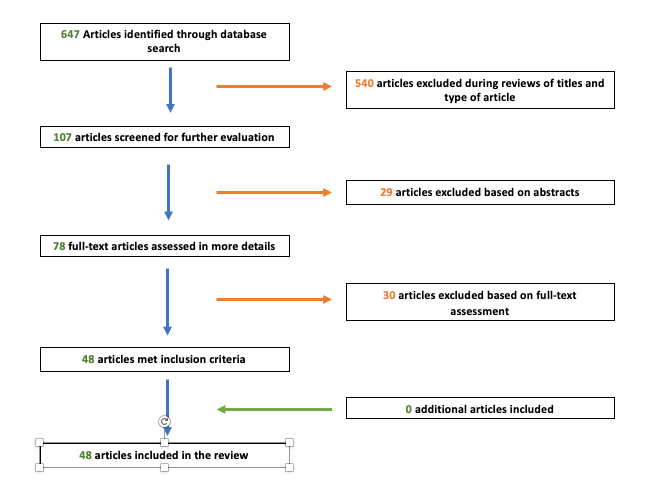


Spanish and Portuguese Data Extraction Flowchart (Latin America Region)


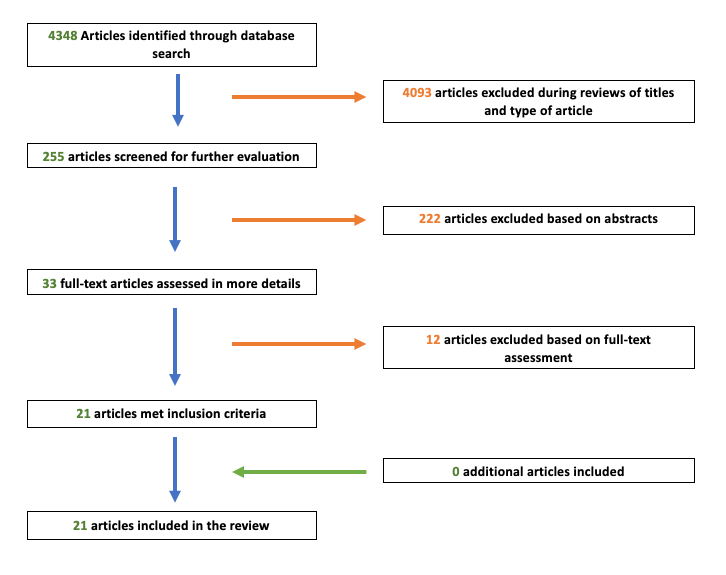


French Language Data Extraction Flowchart


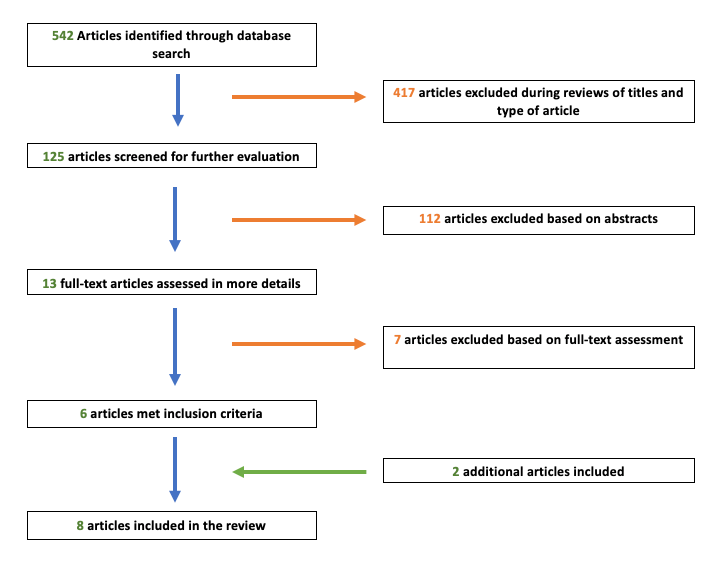

Supplement: S4 File — (DOCX) [file pgph.0002320.s004.docx]
