# Supplementary material for: A rapid qualitative methods assessment and reporting tool for epidemic response as the outcome of a rapid review and expert consultation
Source: PLOS Glob Public Health. 2023 Oct 27;3(10):e0002320. doi: 10.1371/journal.pgph.0002320 (PMC10610454; doi:10.1371/journal.pgph.0002320)
Supplement: Attachment — Submitted filename: Response to Reviewers.docx [file pgph.0002320.s007.docx]

**Response to Reviewers**

We are truly grateful to the two Reviewers who carefully read and commented on our manuscript. Their reading, questions, and recommendations have provoked substantial revision that we believe have strengthened the manuscript.

As recommended by the Editors and Reviewer 2, we have undertaken some restructuring of and more rigorous signposting in the manuscript. We did not, however, move the RQM Assessment and Reporting tool to the Discussion because we contend that it is one result of the rapid review and expert consultation. We have provided fuller justification below, in our point-by-point response to each comment, question, and recommendation. Please note that any line reference refers to the TRACK CHANGES version.

Sincerely,

Tamara Giles-Vernick

| Reviewer 1 | |
| --- | --- |
| Reviewer #1: Very interesting paper with a clear methodology. The study selection process included in the review is well documented. The conclusion is also appropriate. I learned a lot from reading this article. It was a privilege to be among the first readers of this rich and scientifically valuable work. Congratulations to all the authors.  A total of 114 studies were included out of the 8,405 initially identified. Indeed, the exclusion criteria were well described and the study selection flow chart is clear. | Thank you very much for your valuable and insightful comments. Your feedback has greatly contributed to the improvement and refinement of our research. We genuinely appreciate the time and effort you invested in reviewing our work and providing thoughtful suggestions. |
| Question 1 (Minor): Does the high proportion of articles eliminated (98.6%) suggest another study limitation, given that the studies excluded could reveal a greater diversity of objectives and methods used when conducting qualitative researchs during outbreak ? | Thank you for raising the question. The reason that those articles were excluded from the review was that the initial search generated a large number of publications that did not meet our inclusion/exclusion criteria.   It is normal to have a high proportion of articles eliminated during the screening process of a systematic review. For example, a rapid review on a similar topic was published in 2017 (*Reference 3: Johnson G, Vindrola-Padros, C. Rapid qualitative research methods during complex health emergencies: A systematic review of the literature. Soc Sci Med. 2017;189:63-75*.). In that review, 1444 articles were identified through the initial search, but only 22 articles were retained in the final review, resulting in approximately 99.98% of article elimination. Therefore, we do not consider this a study limitation. |
| Question 2: The study selection flow chart shows 12 studies that were added at the end of the selection process (114 + 12 = 126). This suggests that these 12 studies were not subjected to the same selection criteria as the other studies. What was so different about these 12 that they were not evaluated like the other studies? What criteria were they subjected to before inclusion in the study ? Maybe a few details in the methodology might spare readers from asking this question. | We apologize for any confusion. In lines 123-128, we explained the source of these additional studies.  The 12 studies were identified through manual searches of unpublished documents from academic communities, such as the *WHO Social Science Community of Practice*, social sciences network sites, such as *Medical Anthropology Switzerland*, and other professional and technical websites *Relief Web, UN websites, government publication websites, and NGO websites*. Not all relevant literature, especially unpublished documents, government publications, NGO reports, and other grey literature, is included in academic publication databases. Therefore, it is always essential to search these websites to exhaust the literature that needs to be included in the review.    The 12 studies underwent the same selection criteria as the other studies. They were assessed based on our inclusion/exclusion criteria before being finally included in the review.    To clarify the search strategy and the additional web search sites, we have made a few modifications to line 144-151 and added them to Table 1 |
| Question 3 (Minor): Krippendoff's α coefficient has been reported for inter-encoder agreement. However, nothing was said about how it was calculated. Specifically, was any software used ? Would it be useful to add a table (in the appendix) showing this calculation? | We apologize for the lack of clarity.    To calculate Krippendorff’s alpha, we used an online utility, called ‘ReCal OIR (“Reliability Calculator for Ordinal, Interval, and Ratio data”)’ which can be found here:<http://dfreelon.org/utils/recalfront/recal-oir/>    You may also refer to this *reference: Freelon, Deen. "ReCal OIR: ordinal, interval, and ratio intercoder reliability as a web service." International journal of internet science 8.1 (2013).*    Since the website provides only the results of the calculation but not the step-by-step process, there is no table available to show the calculation.    We have added the information about the online utility and the reference to our manuscript (lines 188-191). |
| Question 4: The research team has suggested a 13-criteria matrix for qualitative studies during emergency context. a) Given their importance and specificity, including the difficulty of collecting funding data, shouldn't human resources and funding criteria be presented separately? b) Concerning the second objective of the study (to propose a matrix for notifying and evaluating rapid qualitative studies in emergency situations), wouldn't it be more comprehensive if the research team suggested a scale score for each criteria and for the 13 criteria as a whole? In its current state, this matrix seems to me to be useful only for notification. But if studies are also to be evaluated, a scale should be suggested. | Q4-a We appreciate the reviewer’s thoughtful consideration of the human resources and funding criteria in our assessment tool (clarified and retitled as: **A Rapid Qualitative Methods Assessment and Reporting Tool for Epidemic Response as the Outcome of a Rapid Review and Expert Consultation**  While we acknowledge the importance and specificity of these criteria, we believe that presenting them together under the category of 'Available resources' is justified for several reasons:    1. Interdependency of Human and Funding Resources: In emergency situations, the availability of human and funding resources often goes hand in hand. The success of a rapid qualitative assessment relies not only on the availability of skilled personnel but also on the necessary financial resources to carry out the study effectively. Separating human and funding resources could overlook the critical interdependency between these two factors in determining the feasibility and practicality of implementing a rapid qualitative assessment.    2. Holistic Assessment of Feasibility: By combining human and funding resources under “Available resources,’ we aim to provide a comprehensive evaluation of the overall feasibility of conducting a rapid qualitative assessment. Considering these factors together allows researchers and practitioners to assess the adequacy of resources in their totality, leading to a more informed decision-making process.    3. Practicality and Efficiency: In the context of emergency situations, time is often of the essence. Presenting human and funding resources separately could complicate the decision-making process and potentially create redundancy in the evaluation. By integrating these criteria, our tool streamlines the assessment process, making it more practical and efficient for researchers and decision-makers in urgent situations.  We have added further justification in lines 521-524.    Q4-b: Thank you for your thoughtful feedback on the second objective of our study. We appreciate your insights, but we believe that the RQM assessment tool has widespread utility as is, and we have indicated our intended contributions to the field in the Introduction and Discussion. The development of a core set of criteria through the process we undertook is a necessary prerequisite for the development of a scale, particularly given the robust literature review and scholarly engagement that went into its development. We refer Reviewer 1 to (Boateng, G. O., Neilands, T. B., Frongillo, E. A., Melgar-Quiñonez, H. R., & Young, S. L. (2018). Best practices for developing and validating scales for health, social, and behavioral research: a primer. *Frontiers in public health*, *6*, 149.)  A scale, however, is not a required outcome. It also requires more than just the development of a common standard; it requires field-based validation, reliability testing, and a uniform standard for application and implementation. The assessment tool that we have developed, in contrast, functions more as a checklist. It is intended to serve as a guidance document for a wide range of users, including program designers, funders, researchers, and evaluators. It will be useful for planning, implementation, and evaluation, and it will be of service in providing a standard of quality for common peer evaluation in the field of epidemic response. We also believe that the assessment tool has utility as a policy document for advancing an agenda that further calls for transparency around key criteria for quality RQM. These are all anticipated “next steps” in the life of this guidance tool.  In light of these limitations, we agree that further research should involve a more extensive and diverse group of experts to validate and refine the proposed matrix. |
| **Reviewer 2** | |
| Reviewer #2: Overall comments This is an excellent paper, and I am delighted to have had the opportunity to review it. It gave me a lot of food for thought and will improve my own approaches to RQM. My comments are largely around clarity and structure, as the content itself is very strong. The inclusion of multiple languages in the review is a particular asset of this work. | We thank this reviewer for such a careful and thoughtful read of this manuscript, as well as for critical comments. We are convinced that these questions and suggestions have certainly strengthened the piece. |
| My main editorial point is that I feel the paper is missing the aspect of a discussion that typically contextualises the findings in the literature and really underlines the contribution. The focus on the evaluation matrix is important, but I was left wanting a reflection on how the development of these criteria moves the field forward relative to what we knew before. You make a lot of novel points and I would like to see these shine through more. | We agree with Reviewer 2 that the paper needs to contextualize better its findings and to underscore its contribution. To that end, we have added text in the Introduction (lines 87-92) to clarify the intended uses, target audiences, and applications of our contributions, and the context in which we would like to see these innovations advanced. We have also added in the Discussion section (Lines 435-444) fuller explanation of these contributions. |
| Something that I was consistently unsure about across the paper was about duration of studies – clarity throughout about whether the end of a study was reporting/ end of fieldwork would be very helpful | Reviewer 2’s concern regarding Timeframe is well noted. As we indicate in the Methods section and “Timeframe of the Study” subsection, there was a lack of clarity in the included studies about whether the RQM study timeframe included data collection and reporting, or if it included inception to publication. It remains unclear, however, that stipulating a common standard in this matter will be benefit RQM researchers, because RQM researchers used a wide diversity of research implementation models.  We would thus argue that more transparency is needed, but we would also suggest that it remains unclear whether more consistency is needed. For example, some researchers were funded to undertake discrete research activities, whereas while others were adjacent to intervention programs (ex. hospitals) that had ongoing data collection activities under way. In future research, researchers may benefit from additional guidance and clarity in this matter. |
| On line 79 you say this expands on prior research, but it would be useful to have more about what this prior research found. I suggest you add more detail on what is already known in the existing methodological literature (e.g. a summary of Johnson and Vindrola-Padros’ 2017 review). | We have added a short summary of Johnson and Vindrola-Padros’s 2017 review on lines 82-84, and in keeping with Reviewer 2’s recommendations, signpost where the present manuscript builds on and extends their work. |
| Line 117 – what was the previously published study that you used to replicate the search terms? Could you say more about what it was not possible to replicate the resource extraction? | The previously published study is reference 3: Johnson G, Vindrola-Padros, C. Rapid qualitative research methods during complex health emergencies: A systematic review of the literature. Soc Sci Med. 2017;189:63-75.  We have added an in-text citation and have explained more clearly in the text the limitations we encountered (Lines 137-141).    We were unable to replicate the search terms mainly for three reasons:    1. Not all the literature databases that Johnson & Vindrola-Pados used were available to our research team when the review was conducted. For example, Johnson & Vindrola-Pados searched MEDLINE, CINAHL Plus, Web of Science, ProQuest Central, while we used PubMed, Medline, EMBASE, and Web of Science for English literature.    2. Johnson & Vindrola-Pados only provided one “sample search strategy” in the appendix of their publication, which does not seem replicable across all literature databases due to their varying constructions. (You may download their Appendix and take a look at the sample search strategy:<https://www.sciencedirect.com/science/article/pii/S0277953617304628?via%3Dihub#appsec1>)    3. Johnson & Vindrola-Pados only included English publications, while we expanded the search to include other languages as well. Due to the diverse constructions of literature databases in different countries, it was not feasible for us to use their search strategy across these databases. |
| - Why limit the Spanish/Portuguese sources to Latin American countries – why not do a global search to capture sources published in Spanish or Portuguese from Europe as well? You mention this as a limitation, but it would be useful to acknowledge it in the methods as well. | We agree that it would have been preferable to do a global search capturing sources published in Spanish and Portuguese from Europe as well. Nevertheless, the Spanish-Portuguese research team contended that because this exercise had never been previously conducted in Latin America, and team members concluded that the inclusions would be too voluminous to conduct in a rapid review.  We have included explanation of this choice Lines 130-131 and in Table 1 (Line 134). |
| Could do with a bit more detail in the section on coding, analysis and synthesis – the process seems robust but it isn’t 100% clear how you came to the themes. It would help to know more about how you approach the initial data extraction – i.e. how did the core methods subgroup come up with the initial coding template – and how did you decide the themes through group discussions? Basically, I’d like to really understand how you moved from the literature review through the criteria of RQM quality. - | We apologize for the lack of clarity. We have added additional detail in the text (Lines 200-210). This data extraction sheet (Supplementary file 2, “First round data extraction template for rapid qualitative methods”) was initially developed by the six core Methods subgroup members to extract data on the development, variable contexts, implementation, and impact of RQMs in epidemics. Our 25-point extraction sheet was also shaped by numerous demands for high-quality RQMs in the 2020-2021 pandemic context, for instance, a tool’s capacity to identify vulnerabilities or to be repurposed in other contexts.”    The core Methods subgroup members convened weekly online and piloted the data extraction form repeatedly with sample articles. After discussions, they reached a consensus on the coding template.    “To extract data from the included articles, teams used a pre-defined template developed by the six core Methods subgroup members (Supplementary file 2). The core group was aware of the 10-point data extraction template used for the 2017 review, it developed a 25-point template that would extract data on the development, variable contexts, implementation, and impact of RQMs in epidemics. Clearly, the numerous demands for high-quality RQMs in the 2020-2021 pandemic context also shaped the group’s attention to particular data extraction points, for instance, a tool’s capacity to identify vulnerabilities or to be repurposed in other contexts.” |
| Can you clarify the ‘duration of research’ table - Does ‘duration of research’ relate to the total study time (i.e. from conception to reporting) or to the duration of fieldwork? | We apologize for the confusion.  We have updated the table to accurately reflect this by renaming it as “Timeframe of Research Duration of data collection”. Please note the previous comment regarding research Timeframe. |
| Results: 204 – can you clarify ‘completed’ – does this mean reported/published? | We apologize for the confusion.  Here “completed” means research activities were completed.  We have amended the sentence by explicitly indicating “research activities were completed in less than six months” (lines 241-242). |
| 249 – when you say tools, do you mean the specific method (i.e interview), the way of doing the method (i.e. telephone/video interview) or the resources informing the data collection (i.e. topic guide)? | By “tools”, we refer to the specific research methods, but it is worth noting that there is a lack of clarity regarding what constitutes an RQM “tool” vs an RQM “method”, as the reader has specified. We contend that clarifying this distinction is an important next step to improve RQMs, but we cannot resolve that question in this manuscript.  To make the paragraph clearer, we modified it as follows: “Although most sources described their qualitative data collection methods, only 17% (n=21) published the specific details of the methods used (17, 18), such as the process of data collection or the interview guides. Publishing these specific details not only allows readers to gain insight into the types and framing of questions asked, but it can also save time by facilitating speedy tool selection and adoption and can help promote replicability of the study design. (6, 19).” |
| 257 – why ‘possibly’ in rural regions? Because this wasn’t clearly reported? | Thank you for catching this point. After reviewing our sources from studies in high-income countries, we realized that this point slipped into our analysis from other experiences, not the rapid review. Hence, we have taken out the reference to rural regions. |
| In section ‘C- analytical approaches’ the 2nd and 3rd paragraph repeat some of the same information. This could be condensed. | Thank you for pointing out this repetition, which likely resulted from using Track Changes to revise the manuscript. We have now modified the two paragraphs as follows:  “We cannot clarify different analytical approaches because sources, including peer-reviewed ones, described their analytical approaches in such a way that they either lacked clear definitions of their data analysis approaches or there appeared to be substantial overlap in these approaches, making it nearly impossible to distinguish one from another. As a result, preferred analytical approaches could not be counted.” |
| The box ’10 observed strategies for making rapid qualitative methods ‘rapid’’ only has 9 points | This omission has been remedied. Please see Figure 1, corrected from Table 5. Item 10 was “Digitized Data Entry and Analysis” |
| Evaluation matrix - It is a useful summary of different aspects of RQMs, but I think it would be helpful to state its purpose more clearly. I interpreted that the table is meant as a way to a) inform design of quality studies using RQMs and b) to assess the quality of other RQM studies. I wasn’t 100% sure though, so be really specific about this. | We thank the Reviewer for these comments. Upon review, we determined that “Evaluation Matrix” was not appropriate because we intend for the tool to be used for more than just evaluative purposes: it should be used for research design, implementation, evaluation, and research dissemination purposes. We have therefore changed the title to Rapid Qualitative Methods Assessment Tool.  We respectfully disagree, however, that the RQM Assessment Tool should be moved to the Discussion. It is a specific outcome of our data collection and analysis process. It is therefore more appropriately situated in the Results section. |
| - This section could also from some signposting, as initially I thought it was part of the results. Perhaps the way to address this is to move this section entirely into the discussion. | We have modified the title to improve clarity about intended use, and clearly signposted the intent of the Tool in Table 7 |
| As a further point – is it a matrix? Earlier in the paper you refer to it as a framework, but I’m not sure if is that either. It is a set of criteria that indicate quality. Think on this as a writing team and come up with a consistent way to describe the table | Again, we are grateful to the Reviewer for noting the slippage in our terms, from matrix to framework. As noted above, we agree that this tool is not a matrix, nor is it exclusively for evaluation. We have changed language and signposting throughout to reflect the change from Evaluation Matrix to Rapid Qualitative Assessment Tool. |
| Discussion - As mentioned above, I would recommend bringing the evaluation matrix into this section, with a clear introduction signposting that this is the research team’s interpretation and repurposing of the findings of the review (linking back to what I said re: methods – how did you get to this point through your analysis?) | Again, we respectfully disagree with Reviewer 2, although we appreciate their suggestions and reflections. By situating the RQM Assessment Tool belongs in the Results because it was produced from the Rapid Review.  Please note that in the Methods section (lines 200-201), we have also provided additional description of how we developed the Rapid Qualitative Methods Assessment and Reporting Tool from our analysis. |
| 397 – could you clarify if the timeframe of 4 months for conception to analysis also includes reporting? | We apologize for the confusion.  It should read “...from several days to four months, covering conception to data collection”. We have modified the sentence. |
| In the discussion of training, worth reflecting on skills needed for collecting/analysing data other than interviews/observations. | Thank you very much for your suggestion. We added one sentence to discuss additional skill sets needed for RQM.  Lines 474-476: “Strong skills to observe nuanced or non-verbal communication during community engagement and data collection, to build a rapport with participants, and to conduct reflexive analyses to pinpoint research biases and assumptions are all essential for RQMs.” |
| As above, in the section on ‘applicability to more than one group’ could you clarify what you mean by ‘tools’? | We apologize for the confusion. We have modified the paragraph to clarify the meaning of “tools” as follows: “Standardized data collection and analysis protocols, if available, can be used and adapted to local populations to enhance comparability of RQM data and to optimize the speed and efficiency of research design, data collection, and analysis. Such protocols, when structured, are particularly useful in situations where non-social scientists wish to conduct qualitative research, or where social sciences capacity was limited. Actors with local knowledge should inform and lead design, develop, and adapt and pre-test pre-existing tools to ensure local linguistic, cultural and contextual relevance.  Volkmann demonstrated that when specific tools were available for consultation and were adapted to specific settings, they could ensure rapidity, rigor, and replicability of the study in diverse contexts (41). During epidemics, sharing RQM data collection and analysis protocols through publications, repositories, and other distribution methods is crucial for enhancing collaboration and promoting broader use of these tools.” |
| I agree with the point about the usefulness of RQM in epidemics for exploring vulnerability, but I’m not sure this would be considered a methodological marker of quality for all studies, as it surely depends in part on the questions being asked. Is there a way of caveating this point slightly to reflect this? | Thank you for this suggestion, with which we agree. Not all studies would necessarily explore vulnerability. We have modified the paragraph by rephrasing the second sentence as follows:  “It is essential to pay attention to specific vulnerabilities that are contextually sensitive without assuming beforehand which social groups might be marginalized or excluded. However, the usefulness of RQM in examining vulnerability may vary depending on the research questions being addressed.” |
| Lines 475-476 – I think this is a comment from someone in the writing team, and I agree with it! | The reviewer identified a misplaced comment from one of our reviewers, and we apologize for that. We agree that identifying RQM innovations is needed and desirable. Nevertheless, this is part of the next steps that we will address with the WHO in the future, in part by leveraging the RQM Assessment Tool that we have developed here. |
| Line 488 – this is first mention of ‘the expert committee’, who was this? The writing group? Someone else? If so this should be clearer in the methods section. | We agree that this phrasing is unclear and have specified that we are referring to the entire Methods subgroup. |
| Line 516 – ‘Desclaux’ is named rather than presented as a numbered reference | Thank you for catching this error. We have corrected the reference to refer correctly to the specific source. |
| I would also expect a discussion to include a return to the aims of the paper and to summarise the contribution of the paper relative to the existing literature. | We agree and have added several lines (443-454) in the Discussion to reflect these contributions. |
| - I was interested to see the variation in duration of research across studies (how did 18 studies complete the work in less than a week!?). Did you notice any different in reporting/ methodological congruence between the shorted and longest rapid studies? Does there seem to be an ‘optimum’ duration? | Unfortunately, this question cannot be answered. There is insufficient presentation of research methods timeframes in the selected studies for us to be able to quantitatively analyse the studies, or compare their relative efficacy based on timeline. This will require future meta-analysis. |
| Was there anything about emotional impact on researchers of rapid research? It strikes me that there are different pressures involved in conducting RQM, potentially, e.g. researchers who may end up doing multiple emotionally challenging interview in rapid succession. If not mentioned in the literature, something to reflect in in the discussion. | RQM research in epidemics is taxing for researchers, just as all response activities are taxing for epidemic responders. There is a robust literature on the experience of working in epidemics. However, our selection criteria excluded reflections, memoirs, auto-ethnographies by researchers, and we did not capture these results. Reviewer 2 has raised a fascinating line of inquiry for future research, but our sources did not address the emotional impact of RQMs on researchers themselves. |
| For me a really key finding is that RQM can both be good at accessing ‘hard to reach’ groups and ‘low-hanging fruit’ groups, i.e. professional stakeholders. So this suggests that the positionality of the research team is a really important part of RQM, as who they have immediate access to will likely form the study participants. Again, if not discussed explicitly in the literature perhaps something for the discussion. | This observation is well-noted. However, our review process did not specifically analyze the positionality of specific research teams. Our proxy variable to ensure the inclusion of diverse research teams was the language-based and region-based search approach. This would be a suitable subject for future meta-analysis. |
| Typos Line 139 – in exchanges with funding? Line 165 – a key limitation is the lack… Line 306 – others used open-source materials for analysis Line 493 – not sure what this means – ‘wrote analytical memos to adjust those included’ Consistency of spelling – gray/grey | Thank you very much for pointing out the typos and errors. We have corrected them as you suggested.  We chose to use “grey literature” throughout the manuscript. |
